# Comparison activity of pure and chromium-doped nickel oxide nanoparticles for the selective removal of dyes from water

**DOI:** 10.1038/s41598-024-53490-6

**Published:** 2024-02-18

**Authors:** Zahraa H. Athab, Ahmed F. Halbus, Sura Bahaa Mohammed, Abbas J. Atiyah, Hussein Idrees Ismael, Nahlah Salman Saddam, Sadiq J. Baqir, Hasan F. Alesary, Sameer Algburi, Nadhir Al-Ansari

**Affiliations:** 1 Environmental Research and Studies Center, University of Babylon, Hilla, Iraq; 2 Department of Chemistry, College of Science, University of Babylon, Hilla, Iraq; 3 College of Engineering, University of Babylon, Hilla, Iraq; 4 Almustaqbal University College, Babylon, Hilla, Iraq; 5 Department of Chemistry, College of Science, University of Kerbala, Karbala, Iraq; 6 College of Engineering Techniques, Al-Kitab University, Kirkuk, 36015 Iraq; 7 Department of Civil, Environmental and Natural Resources Engineering, Lulea University of Technology, Luleå, Sweden

**Keywords:** Cr/NiONPs, Selective removal, Adsorption, Reactive red 2 dye, Crystal violate, Environmental sciences, Engineering

## Abstract

The current study involves a synthesis of a composite of nickel oxide nanoparticles (NiONPs) with a chromium dopant to yield (Cr/NiONPs). Synthesis of nickel oxide was performed by the co-precipitation method. The synthesis of the composite was conducted by the impregnation method. FTIR, EDX, SEM, and XRD were used to characterize the synthesized materials. The synthesised materials’ point zero charges (PZC) were performed using the potentiometric titration method. The obtained results show that the PZC for neat nickel oxide was around 5, and it was around 8 for Cr/NiONPs. The adsorption action of the prepared materials was examined by applying them to remove Reactive Red 2 (RR2) and Crystal Violate (CV) dyes from solutions. The outcomes demonstrated that Cr/NiONPs were stronger in the removal of dyes than NiONPs. Cr/NiONPs achieved 99.9% removal of dyes after 1 h. Adsorption isotherms involving Freundlich and Langmuir adsorption isotherms were also conducted, and the outcomes indicated that the most accurate representation of the adsorption data was offered by Langmuir adsorption isotherms. Additionally, it was discovered that the adsorption characteristics of the NiONPs and Cr/NiONPs correspond well with the pseudo-second-order kinetic model. Each of the NiONPs and Cr/NiONPs was reused five times, and the results display that the effectiveness of the removal of RR2 dye slightly declined with the increase in reuse cycles; it lost only 5% of its original efficiency after the 5 cycles. Generally, Cr/NiONPs showed better reusability than NiONPs under the same conditions.

## Introduction

In our modern life and the industrial revolution, massive volumes of harmful substances are constantly released into the environment^1–3^, such as phosphate, heavy metals, biological pollutants, nitrate, sulphate, and acidic rain^4–6^. Among a wide range of these pollutants, synthetic dyes are utilized in several kinds of applications and industries like plastics, paper, textiles, leather cosmetics and dyeing industries^7–9^. Due to the deep color and rigid aromatic structure of these dyes, color stuff that is discharged from these dyes can lead to hazards and can show environmental impact^10,11^. In general, the presence of dyes in water can cause changes in both of physical and chemical properties of stream^12,13^, underlying and lake water, these are reduction of oxygen content for the polluted waters, affecting the range of penetration for sunlight into waters; retarding photosynthesis and interfering with gas solubility in water bodies^14–16^. There are several kinds of polluted dyes, and in this context, textile synthetic dyes are a major class of these polluted dyes^17,18^. One significant sort of these dyes appears to be azo dyes, which are classified based on whether or not the molecule contains azo bonds (–N=N–)^19^. These dyes consist of triazo, diazo, and monoazo^20^. Due to their rigid structure, these dyes are resistant to sunlight and oxidation agents, so it is impossible for these dyes to be fragmented into inorganic mineral materials under natural, traditional techniques of anaerobic conditions^21^. Therefore, it's imperative to develop a practical strategy for eliminating these contaminated dyes from the environment. These include adsorption processes and photocatalytic reactions^22,23^. In this context, adsorption of the polluted dyes can be conducted in the existence of different adsorbents^24,25^, and metallic oxides are an important type of this adsorbent^26^.

**Figure 1:**
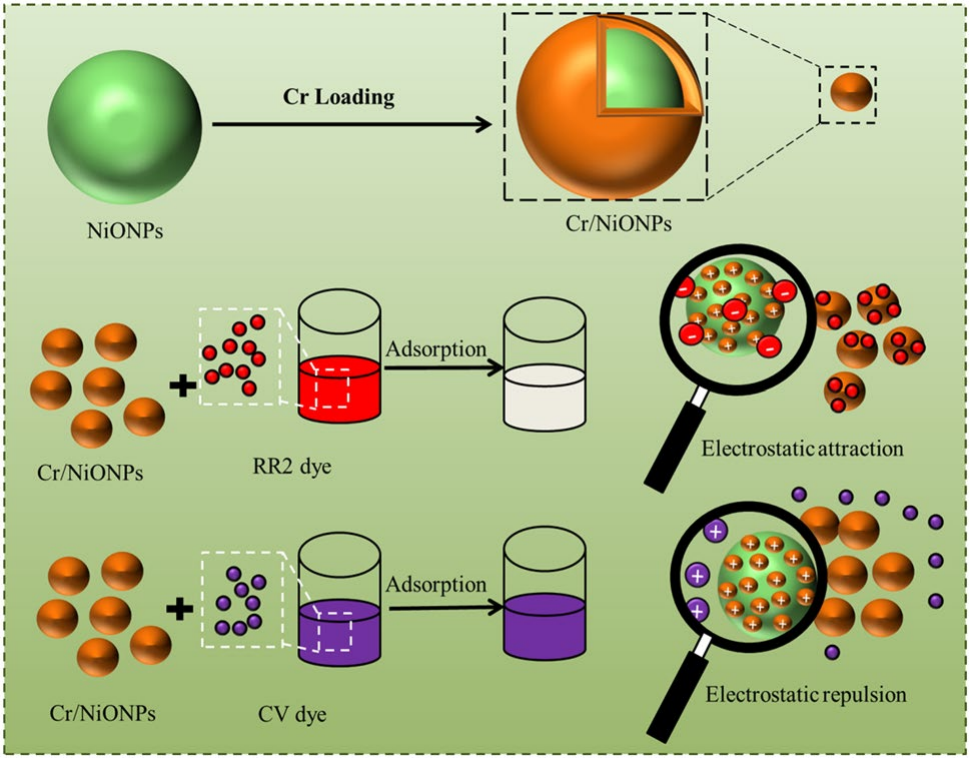
Schematic illustration of the synthesis of Cr/NiO composite nanoparticles and its use for selective removal of anionic dye RR2 dye by adsorption via electrostatic attraction and shows the lack removal of cationic dye CV dye.

Generally, a variety of adsorbents can be employed to achieve this goal. In this context, metallic oxide semiconductors were utilised as efficient adsorbents because of their superior chemical and physical properties^26,27^. Among these different types of semiconductors, nickel oxide (NiO), this oxide exists in a deformed form because of the existence of excess oxygen atoms, and this leads to the formation of holes among the neighbouring ions of Ni^2+^ ions, thus lead to the oxidation of nickel ions from (Ni^2+^ to Ni^3+^). Generally, changes in the color of this oxide result from this process^28^. In heterogeneous photocatalytic systems, nickel oxide can be employed singly or in combination with a supported co-catalyst. Among different catalytic applications of this oxide, it can be used as an adsorbent to remove a wide range of pollutants from their environment^26^. Many efforts were directed to improve the catalytic activity of NiO. These include enhancement of surface and electronic properties like metal/nonmetal doping, dye-induced photosensitization and secondary semiconductor coupling^29,30^. Due to their enhanced magnetic qualities and potential applications in a variety of industries, such as memory storage, sensors, and catalysis, there has been a rise in interest recently in synthesizing magnetic nanoparticles of Ni, Co, and Fe. In medicine, they are utilized for magnetic resonance imaging, magnetically regulated drug delivery, and the treatment of cancer cells by heating them to a high temperature^31–33^. Numerous chemical and physical techniques, such as co-precipitation^34^, sol–gel^35^, microemulsion^36^, hydrothermal reaction^37^, electrospray synthesis and laser ablation^38^ are used to synthesise NPs. These methods can effect on the structural properties of formed NPs. It is still a big challenge to improve the catalytic action of metal oxide by doping some reactive metals. Doping of NiO with metals is used effectively in a wide range of applications. Among different methods to reach the goal, metal doping seems to be an excellent way to improve its activity. It is found that doping MO with metals can lead to substituting a small fraction of Ni in a NiO lattice with another metal action, this is known as the doping process, and it could be an approach to enhance NiO catalyst performance. It is believed that metal doping can change the chemical bonds at the surface of NiO; in this manner, O_2_ vacancies that are advantageous to the catalytic reaction can be produced by metal doping, which can cause lattice imperfections. Metal oxide doping with metals can affect different scenarios like electronegativity, ionic radius, oxidation state on the redox ability of Ni-based and physicochemical characteristics^39–41^. Generally, Cr doping into a NiO lattice can increase NiO catalytic activity by promoting surface lattice O_2_ action improved surface Ni^2+^ concentration and active O_2_, enhancing the level of catalytic activity^42,43^. Hu et al.^44^ noted that NiONPs exhibited a high capacity for adsorption as well as a high rate of adsorption toward congo red. Hu et al.^44^ studied the kinetic parameters and isotherm for the adsorption of dye on NiONPs and revealed that NiONPs have excellent adsorption capability with congo red. SiO_2_ with NiO were utilised as effective adsorbents for removing CR from wastewater, according to Lei et al.^45^, who reported that they had an adsorption capacity of 204.1 mg/g. Furthermore, Shao and Huang^46^ investigated the adsorption of anionic dyes like CR, methyl blue, and methyl orange on NiONPs. They discovered that NiONPs are an effective adsorbent for the removal of dye from aqueous solutions. Although the superior adsorptive properties of NiONPs towards organic dyes, no work till now was performed to study reactive red 2 and crystal violate dyes adsorption onto NiONPs and Cr/NiONPs. Therefore, the main goal of the current study is to evaluate how well reactive red 2 and crystal violate dyes are removed by adsorbent NiONPs and Cr/NiONPs. The current work would involve modification properties of nickel oxide by doping with chromium to yield Cr/NiONPs. The activity of the synthesised materials would be screening by investigation removing of Reactive Red 2 dye and Crystal Violate dye from its aqueous solutions by adsorption over these prepared materials as shown in Fig. 1. We also report here the use of NiONPs and Cr/NiONPs, for the separation and selective adsorption of cationic CV and anionic RR2 dye. Despite the ubiquity of NiONPs and Cr/NiONPs, there have been very few reports on selective dye separation and no reports on the use of NiONPs and Cr/NiONPs for RR2 and CV dye removal.

## Materials and methods

### Materials

Every chemical utilized in this work was extremely pure. Chromium nitrate nonahydrate Cr(NO_3_)_3_·9H_2_O and nickel nitrate hexahydrate Ni(NO_3_)_2_·6H_2_O were obtained from Sigma-Aldrich with a purity of 99.9 and 97.9%, respectively. Anhydrous sodium carbonate was obtained from Sigma-Aldrich with a purity of 99.9%. Crystal Violet dye supplied by Merck has the molecular formula of C_25_H_30_N_3_Cl, a molecular weight of 407.98 g/mol and it has a maximum absorbance of 580 nm. Reactive Red 2 dye C_19_H_10_Cl_2_N_6_Na_2_O_7_S_2_, molecular weight 615.3 g/mol was supplied from Merck, it has maximum absorption at 541 nm.

### Synthesis of NiONPs

NiONPs were synthesized using the co-precipitation method and then followed with thermal treatment of the precipitate. Nickel nitrate hexahydrate, 5.0 g was dissolved in 500 mL of deionzied distilled water in a beaker of 1.0 L. This solution was stirred continuously under atmospheric conditions at 333 K. Then 0.1 M sodium carbonate was added gradually drop by drop to raise the pH of the solution to reach pH 8.5. Then, this solution was kept under the same conditions for another two hours in order to ensure the completion precipitation process. The obtained light-green solution was separated by filtration under a vacuum by washing with deionised water five times to eliminate any impurities. Then, the produced solid material was dried at 373 K for four hours in a drying oven. After that, it was calcined at 500 ºC for four hours in a furnace^47^.

### Synthesis of Cr/NiONPs

Doping of nickel oxide with chromium was conducted by impregnation method. According to the method, 2 g of the prepared NiONPs was added to a solution of 0.2 g of chromium nitrate dissolved in 40 mL of deionized distilled water and it was stirred at 70 °C for 5 h. Then, the solid materials were separated by filtration and dried overnight at 110 °C, after that, it was washed with deionised distilled water many times to make them free of nitrate, and dried overnight at 110 °C. The process is analogous to the metal-doped titania nanoparticles^48^, but in our case, doping of nickel oxide with chromium was conducted by the impregnation method. This doping has not been reported before for NiONPs. The powder that emerged was then burned for 5 h at 300 °C. Finally, the obtained materials were designated as Cr/NiONPs.

### Characterisation of the prepared materials

The prepared materials' crystal structure was examined using powder X-ray diffraction. Surface functional groups of the prepared materials were probing using FTIR. The surface morphology of the prepared materials was investigated using SEM instrument JEOL JSM-6700F instrument (Germany). Point zero charge (PZC) for NiONPs and Cr/NiONPs was determined using the potentiometric titration method^49^. According to this method, KNO_3_ (0.03 M, 100 mL) was used as a blank solution, and to this solution, NaOH (1.0 M, 1.0 mL) was added. HNO_3_ (0.10 M) was used to titrate the solution. A mixture of 100 mL of KNO_3_ with 0.2 g of the solid material was stirred for 24 h, then; 1 mL of NaOH was added and titrated with HNO_3_ as it was done with a blank solution. The titration's results are plotted against the mixture's pH as a volume of added acid and the resulting intersection point between two curves is referred to as PZC for the used material^49^.

### Adsorption studies

RR2 and CV dyes were used to check the removal activity and the adsorption capacity of the NiONPs and Cr/NiONPs materials. A UV–Vis spectrophotometer was utilized to assess the study outcomes at 541 nm and 580 nm for RR2 and CV, respectively. The produced nanoparticles were added to dye solutions containing 100 mg/L. The solutions were then continuously shaken for an hour in the dark using a platform shaker. Prior to and following adsorption, the absorbance at the wavelength of maximum absorption was measured. For NiONPs and Cr/NiONPs, where the weight of the NPs was 0.1 g, batch adsorption experiments were carried out to assess the adsorption uptake quantity (Qe) and the percentage of dye removal (R%) of RR2. A 100 mL serial RR2 dye solution (10–60 mg/L) was added to the nanoparticles in isolated conical flasks. The materials were mildly shaken until equilibrium was established using a shaker speed of 150 rpm. Each test was made three times, and the dye concentration was determined using the RR2 dye’s maximum absorbance (541 nm). The R% and the quantity of dye adsorbed per unit mass of adsorbent at equilibrium Q_e_ (mg/g) and at a particular time Q_t_ were determined using the following formulas^7,9^:

$$\% R = \frac{{C_{0} - C_{t} }}{{C_{0} }} \times 100\%$$

$$Q_{e} = \frac{{(C_{0} - C_{e} ) V}}{m}$$

$$Q_{t} = \frac{{(C_{0} - C_{t} ) V}}{m}$$

The initial dye concentration (mg/L) prior to adsorption and the dye concentration at time t are denoted by C_0_ and C_t_, respectively, where C_e_ (mg/L) is the concentration at equilibrium. The m (g) and V (L) denote the dose of nanoparticles and the dye solution volume, respectively.

### Adsorption isotherm

In order to determine the adsorption isotherms for the RR2 dye on the produced nanoparticles NiONPs and surface modified NiONPs, Langmuir and Freundlich isotherms were used. Freundlich adsorption isotherm is applied in a case of physical adsorption that occurs at heterogeneous adsorption sites and it can occur in multilayers over the adsorbent surface (n) > 1^7,9^. This isotherm can be represented in the following relation:

$$\log Q_{e} = \log K_{F} + \frac{1}{n}\log C_{e}$$

where K_F_ and n are Freundlich constants.

The Langmuir adsorption isotherm is applied in a case for adsorption that occurs at a specific homogeneous surface. It can be represented in the following relation^7,9^:

$$\frac{{C_{e} }}{{Q_{e} }} = \frac{1}{{Q_{max} K_{L} }} + \frac{{C_{e} }}{{Q_{max} }}$$

where Q_max_ is the maximum amount of the dye molecules that are adsorbed per unit mass of nanoparticle and K_L_ is the Langmuir constant.

### Adsorption kinetics

The adsorption kinetic models of the adsorption process were established using the pseudo first order and pseudo second order models. These are the models which are most commonly used to explain how organics, including pigments from aqueous solutions, adsorb on various adsorbents. Adsorption experiments were conducted using 100 mL of various dye concentrations in the range of (10–60 mg/L) with 0.1 g of adsorbent in order to examine the RR2 dye adsorption kinetics on NiONPs and Cr/NiONPs. Adsorption experimental data were initially examined in pseudo-first order, and the rate constant (k1) was determined using the equation^7,9,50,51^:

$$\frac{1}{{q_{t} }} = \left( {\frac{{k_{1} }}{{k_{d} }}} \right)\left( \frac{1}{t} \right) + \frac{1}{{q_{e} }}$$

For this relation, qe and qt (mg/g) refer to the amounts of RR2 dye molecules that adsorbed over the used adsorbent at equilibrium and at a time t (min), respectively, and k_1_ is the adsorption rate constant for the first ordered reaction kinetics in (min^−1^). According to Yuh-Shan Ho, The pseudo-second-order rate was found using the following formula^52,53^:

$$\frac{t}{{q_{t} }} = \frac{1}{{k_{2} q_{e}^{2} }} + \frac{t}{{q_{e} }}$$

According to this relation, k_2_ (g/mg min) is the rate constant for pseudo second ordered reaction kinetics.

### The surface area for the synthesized materials

The surface area for the synthesized materials was calculated according to the Sares method^54,55^. according to this method, 0.5 g of materials was acidified with HCl (0.1 M) in 10 ml with adjusting acidity at a pH of 3.0–3.5 using a controlled pH meter. The volume of the mixture was completed to a final volume of 50 mL with deionized distilled water. Then 10.0 g of NaCl was added to this mixture. This solution was titrated with NaOH (0.1 M), in a thermostatic water bath to maintain temperature at 25 °C. A pH of solution adjusted at pH 4. After that, carefully record the volume of NaOH from the burette that is required to raise the value of pH from 4.0 to 9.0. The surface area according to this method was estimated according to the following relation^54,55^:

$${\text{S}}\left( {{\text{m}}^{{2}} /{\text{g}}} \right) = {\text{32V}} - {25}{\text{.}}$$

## Results and discussion

### Characterisation of NiONPs and Cr/NiONPs

#### SEM of nanoparticles

**Figure 2:**
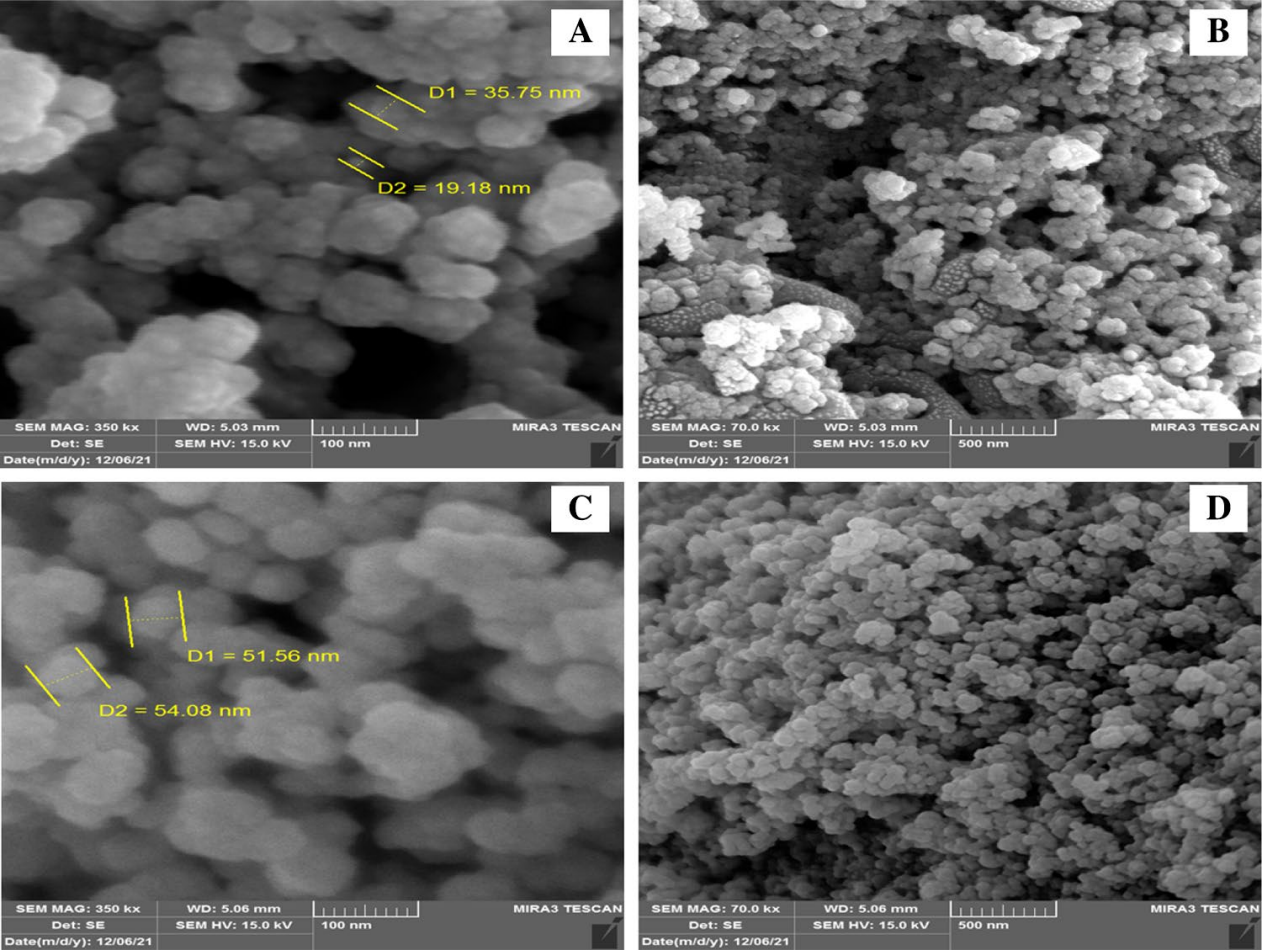
SEM images of (**A**) and (**B**) the NiO nanoparticles, (**C**) and (**D**) Cr/NiO composite nanoparticles at different magnifications and scales.

SEM technique was utilised to probe the surface morphological features of the synthesised materials. Figure 2 shows the SEM images of NiONPs and Cr/NiONPs, from these images it can be seen that NiO nanoparticles almost showed a particle size distribution in the range of 20–75 nm. SEM images for neat NiO nanoparticles are shown in Fig. 2A,B. These particles are relatively homogenously distributed in a spherical shape. In the case of the Cr-doped nickel oxide (Cr/NiONPs), their images are presented in Fig. 2C,D. These images showed that the doped NiONPs with chromium are almost still homogeneous and there was a slight increase in particle size with a slight aggregation for doped oxide in comparison with neat oxide. This aggregation is probably due to electrostatic attraction that is induced due to existing of a Cr dopant at the surface of NiONPs^56^.

#### XRD and PZC of the synthesised neat NiONPs and Cr/NiONPs

**Figure 3:**
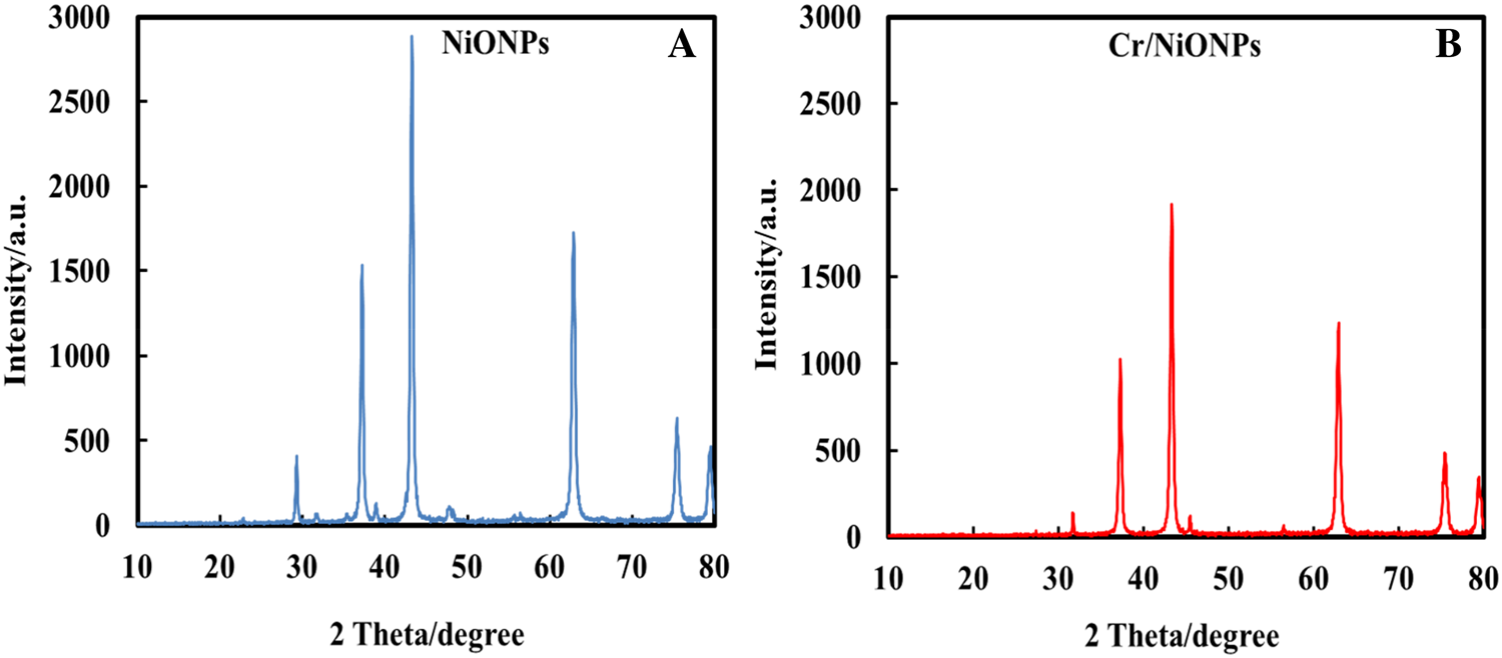
(**A**) and (**B**) XRD patterns of the NiONPs and Cr/NiONPs respectively.

Figure 3A,B display the XRD patterns for the synthesized materials. XRD patterns for NiONPs show the existence of the typical peaks for NiO, in the range of 2θ = 10–80, in accordance with JCPDF file 78-0643. The peaks presented in Fig. 3A are 2θ = 28, 38, 43, 64, 75, and 79, which belong to (400), (111), (200), (220), (311) and (222) planes respectively^57^. XRD patterns of Cr/NiONPs (Fig. 3B) showed almost the same diffraction patterns in comparison with neat NiO. From these patterns, it can be seen a weak peak at 2θ = 33 (202) which belongs to the presence of chromium oxide within a crystal structure of NiO at a small level. Also, it is clear that anther peaks of NiO were incorporated with that of chromium oxide. It can be concluded from these patterns that doping of NiO with chromium almost does not alter its crystalline structure^58^. A small shift in peaks in the case of Cr/NiO in comparison with those of neat NiO indicates the occupation of some nickel sites by chromium ions. So, the replacement of some Ni^2+^ ions and Cr^3+^ ions can lead to a slight shift in the XRD reflections.

**Figure 4:**
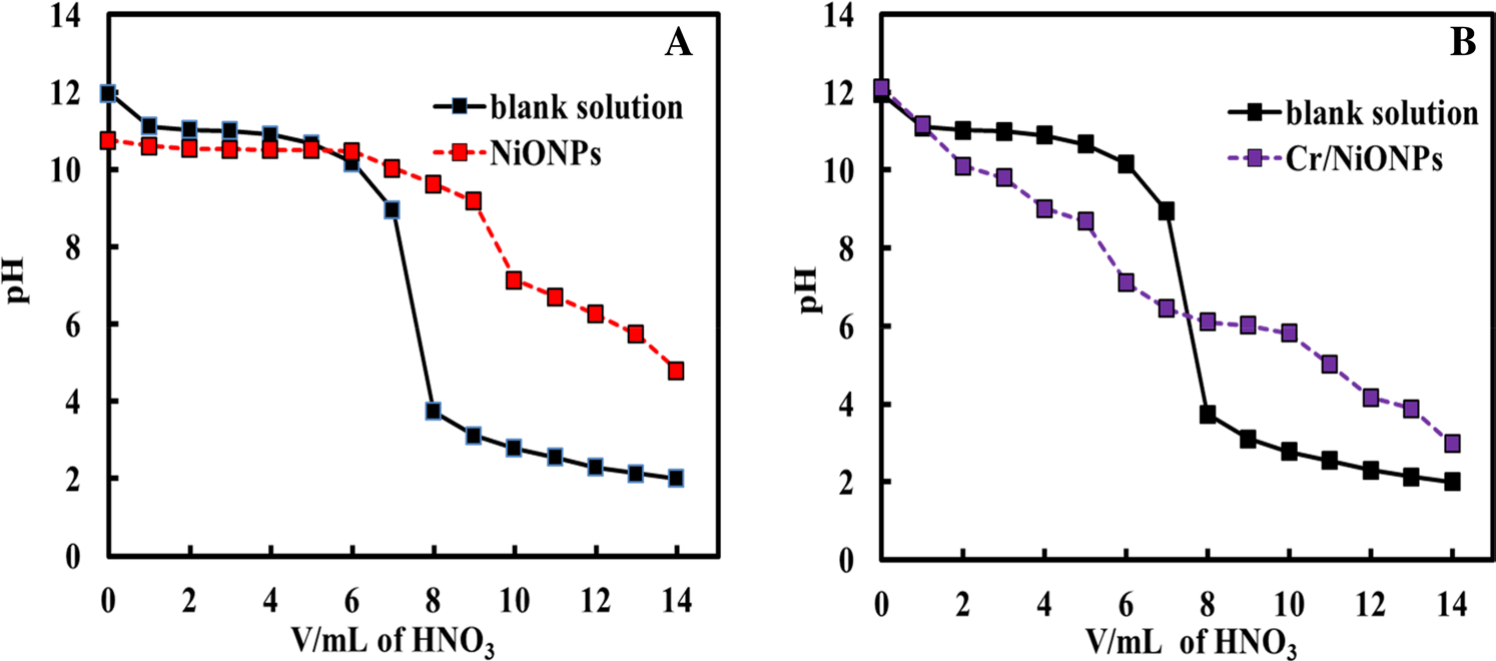
(**A**) and (**B**) PZC of the NiONPs and Cr/NiONPs respectively.

Figure 4A,B display the PZC for neat NiO and Cr/NiO, respectively. The PZC for a material is defined as the pH at which the net charge of the total particle surface is equal to zero (i.e. concentration of H^+^ is equal to OH^−^). From the obtained results, PZC for neat nickel oxide was around 5, and it was for Cr/NiONPs around 8. This means that for neat NiONPs, its surface shows a net positive charge at pH values less than 5 and show negative charge at pH values more than 5. In the case of Cr-doped nickel oxide (PZC = 8), its surface shows net positive charges at pH less than 8 and shows a net negative charge at pH greater than 8. This feature is probably due to the presence of some ionic species such as Ni^2+^ at the surface of doped nickel oxide^59^.

The obtained results of the surface area for the prepared materials were 645 m^2^/g for the free NiO and 724 m^2^/g for Cr/NiONPs. From these results, it can be seen that there was a significant increase in surface area for Cr/NiONPs in comparison with free NiO with chromium species. It was discovered that modification of NiO by doping with chromium dopant can lead to improved textural properties via the replacement of Cr ions instead of Ni ions in the NiO lattice. This leads to increased porosity of this material which leads to an increase in the surface area. So, a higher available active site could be exposed for any desirable reaction^60^.

**Figure 5:**
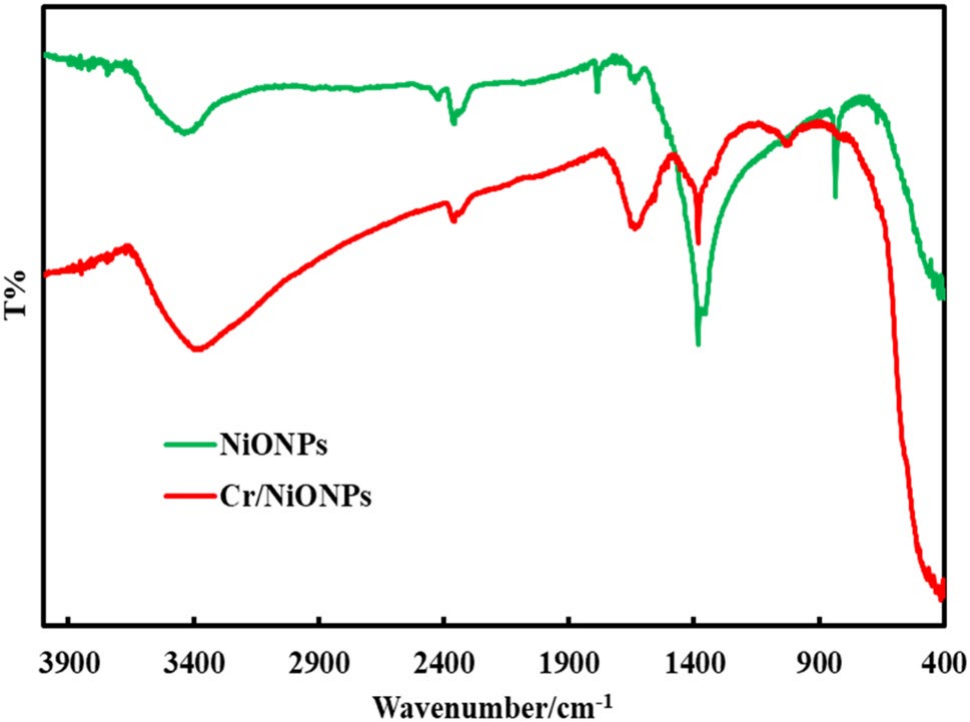
FTIR of neat NiO and Cr/NiO nanoparticles.

The FTIR spectra of NiO and Cr-NiO nanoparticles are displayed in Fig. 5. The broad absorption peak, which can be found at about 1643 and 3450 cm^−1^, displays the bending and stretching vibration of O–H that resulted from water molecules that were adsorbed on the surface of the materials as prepared^43,61^. Moreover, the vibration of the Ni–Cr bonds may be attributed to the two strong bands at 1437 and 1076 cm^−1^ in Cr-doped NiO materials, demonstrating the successful doping of Cr ions in NiO nanoparticles^43^. The stretching frequency for NiO at 483 cm^−1^ corresponds to the Ni–O bond^62^. In addition, bands at 880 cm^−1^ might be the stretching vibration of Cr–O^43^. Consequently, the presence of chromium atoms in the NiO lattice is confirmed by the production of Cr–O. This demonstrates that chromium atom inclusion in the NiO lattice is the cause of the observed shifts in FTIR spectra^43^*.* The bands at 1437 and 1076 cm^−1^ in Cr/NiO materials are attributed to Ni–Cr bond which confirms formation of Cr–Ni bond as it was cited in the reference^43^. The band at 1400 cm^-1^, the bending vibration around 1400 cm^−1^ is attributed to the banding vibration of OH group of the catalyst surface^63^.

**Figure 6:**
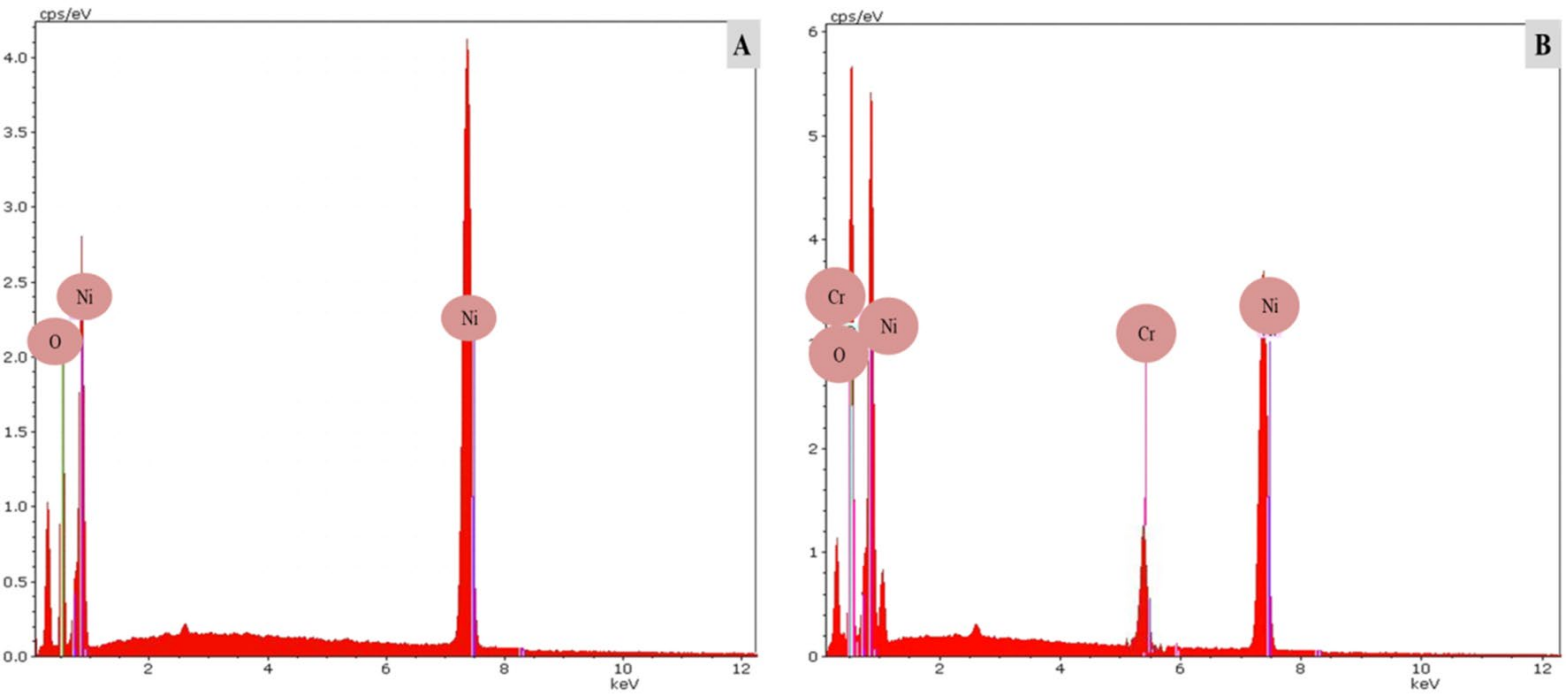
EDX spectrum of the (**A**) NiONPs and (**B**) Cr/NiONPs.

Figure 6A,B shows EDX analysis of the NiONPs and Cr/NiONPs. Figure 6A shows the presence of Ni and O before the treatment as expected. The findings show that the EDX spectra include only Ni and O (Fig. 6A), with no other discernible elemental contaminants. However, the EDX spectrum of the NiONPs after treatment with Cr (Fig. 6B) shows a new peak for Cr. The results confirm the presence of Cr with NiO. EDX analysis confirms the presence of a chromium dopant and it is present as a chromium ion (Cr^3+^). In this work, chromium ion doped with NiONPs was in the form of Cr^3+^ which is in agreement with other studies^64,65^.

### Adsorption of RR2 dye over neat and doped nickel oxide

**Figure 7:**
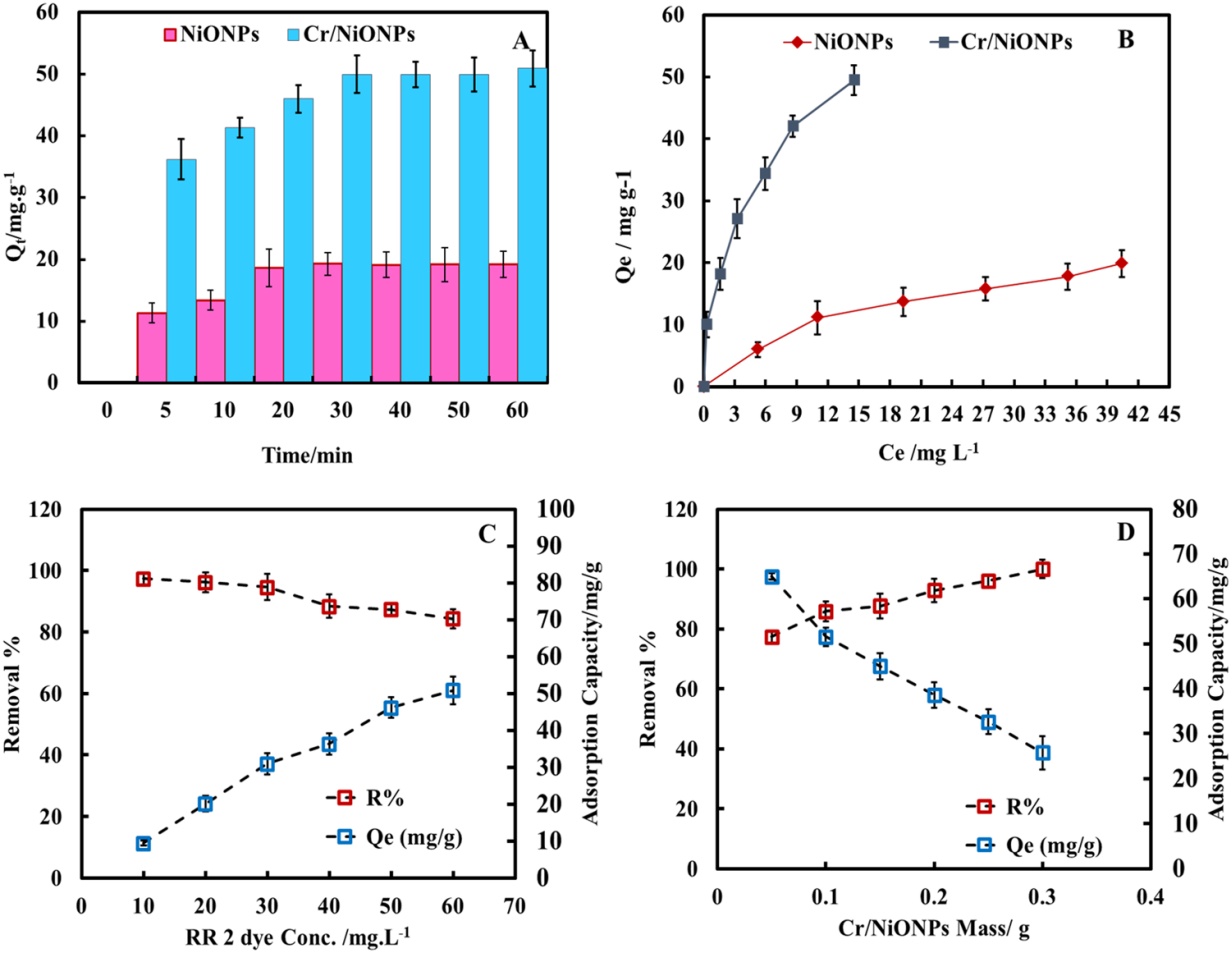
(**A**) The impact of time on adsorption ability of RR2 dye by NiONPs and Cr/NiONPs (NPs mass: 0.1 g in 100 mL of RR2 solutions, the initial RR2 conc.: 60 mg/L, t = 25 °C, at 150 rpm, pH 6). (**B**) Adsorption isotherm of RR2 dye on both NiONPs and Cr/NiONPs (**C**) and (**D**) the impact of RR2 concentration and the mass of NiONPs and Cr/NiONPs on the adsorption capacity and R%.

Aqueous solutions of the RR 2 dye (100 mL, 60 mg/L) were employed to examine the adsorption capacity of the synthesised materials. In each case, 0.1 g of the materials were suspended at room temperature in an atmosphere with regular stirring. Then periodically, a 3 mL centrifuged sample of the reaction mixture was taken. Using a syringe with a long, pliable needle, the obtained supernatant liquid was meticulously eliminated, and the absorbance was determined at 541 nm. The obtained results are presented in Fig. 7A–D. It was revealed that there was a significant rise in the efficacy of RR2 dye elimination by adsorption over Cr/NiONPs more than that in the case of adsorption over neat nickel oxide under the same adsorption conditions. The reason for that can be accredited to the nature of the surface of the adsorbent, as PZC for neat NiONPs and Cr/NiONPs was at pH 5 and pH 8 respectively, so it is expected that the surface of Cr/NiONPs shows a net positive charge more than neat nickel oxide. This would result in the formation of a potent electrostatic interaction among the dye molecules and the surface of the adsorbent because RR2 dye is an ionic dye. This observation leads to an rise in the efficacy of dye removal over the Cr/NiONPs surface in comparison with neat NiONPs surface applying the same adsorption conditions. Figure 7A,B display the effect of adsorption time on the efficiency of dye removal. It is obvious that the effectiveness of dye removal increased with time progress for each neat and doped nickel oxide. This results from the increased availability of adsorption sites with time progress for both adsorbents. The influence of dye concentrations on the efficiency of dye removal over 0.1 g of adsorbents is presented in Fig. 7C. The obtained outcomes exhibited that there was a decrease in the efficiency of dye removal with the increase in the concentration of the used dye. This possibly rises from the limitation of the number of available adsorption sites at the surface of the adsorbent in comparison with the increase in the number of dye molecules. Additionally, a higher concentration of dye causes the solution's density to rise which in turn can affect the rate of dye diffusion with a possible increase in the viscosity of the solution^66^. All these observations can affect negatively the rate of dye mobility in the solution. The effect of the concentration of the used adsorbent (Cr/NiONPs) on the efficiency of RR2 dye removal is shown in Fig. 7D. This study was conducted over a range of masses from 0.05 to 0.4 g, the obtained results showed an rise in the competence of dye elimination as the mass of the used adsorbent was increased up to 0.4 g, under applying the same other reaction conditions. This observation can be accredited due to an rise in the available adsorption places with an rise in the mass of adsorbent. This led to an rise in adsorption ability due to this feature^67^.

**Figure 8:**
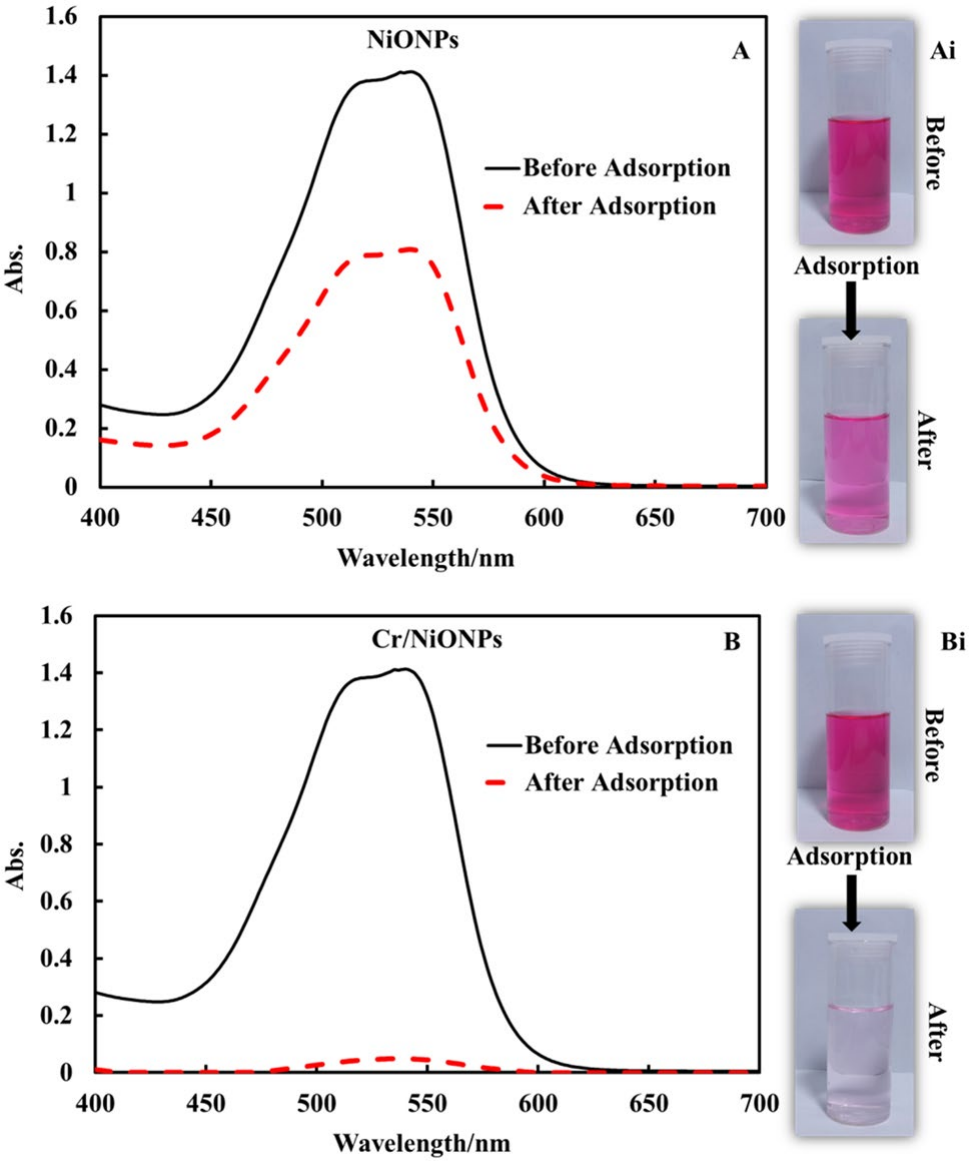
Adsorption behaviors of NiONPs and Cr/NiONPs toward RR2 dye: UV–Vis spectra of RR2 dye prior to and following adsorption by using (A) NiONPs (B) Cr/NiONPs. Photographic images of (**Ai**) NiONPs and (**Bi**) Cr/NiONPs with RR2 dye solutions before (up) and after (down) adsorption.

Adsorption behavior for RR2 over each of NiONPs and Cr/NiONPs is shown in Fig. 8. It is clear that the removal efficiency of RR2 dye over Cr/NiONPs was significantly more efficient than that for adsorption over neat NiONPs. This can be attributed to the nature of the Cr/NiONPs surface that showed a PZC value around 8, this means that there is a positive surface charge. The result of this net positive surface charge is the increase of attraction forces between RR2 molecules and the adsorbent surface^68^.

**Figure 9:**
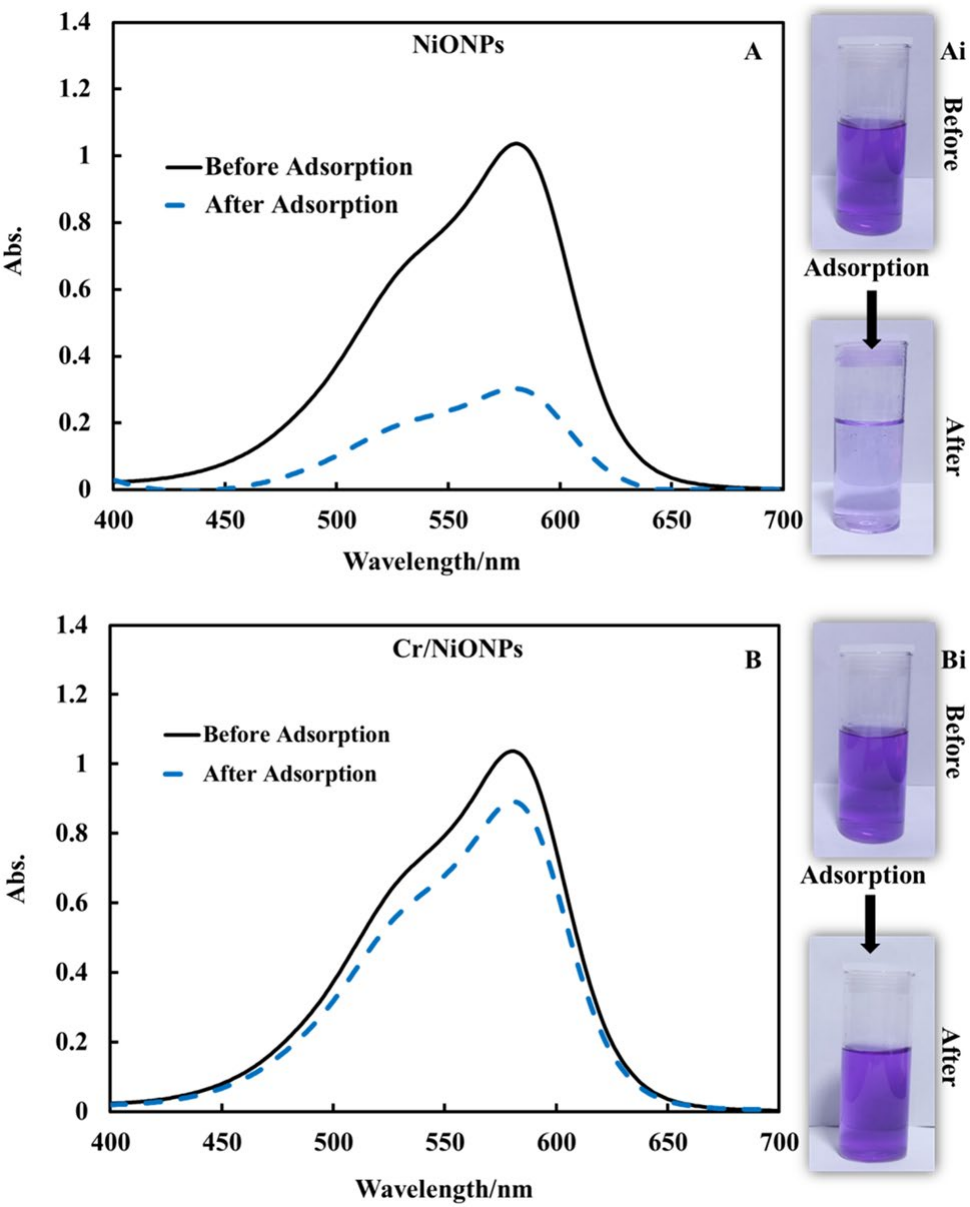
Adsorption behaviors of CV on NiONPs and Cr/NiONPs surface: UV–Vis spectra of CV dye prior to and following adsorption by using (**A**) NiONPs (**B**) Cr/NiONPs. Photographic images of (**Ai**) NiONPs and (**Bi**) Cr/NiONPs with CV dye solutions before (up) and after (down) adsorption.

The effect of net surface charge on the efficiency of dye removal can be further confirmed by following the adsorption of CV over NiONPs and Cr/NiONPs by applying the same adsorption conditions. Figure 9 shows the adsorption behaviors of CV dye with NiONPs (Fig. 9A,Ai) and Cr/NiONPs (Fig. 9B,Bi). In this case, the adsorption pattern of CV dye on NiONPs and Cr/NiONPs was observed to exhibit inverse actions. It was found that the removal efficiency over neat NiONPs was more efficient than that of Cr/NiONPs. This phenomenon is attributed to the nature of CV dye because this dye has acidic groups in its structure. This would affect repulsion and attraction forces that occur between dye molecules and the NPs surface.

### Adsorption isotherms for removal of RR2 dye over neat and doped nickel oxide

**Figure 10:**
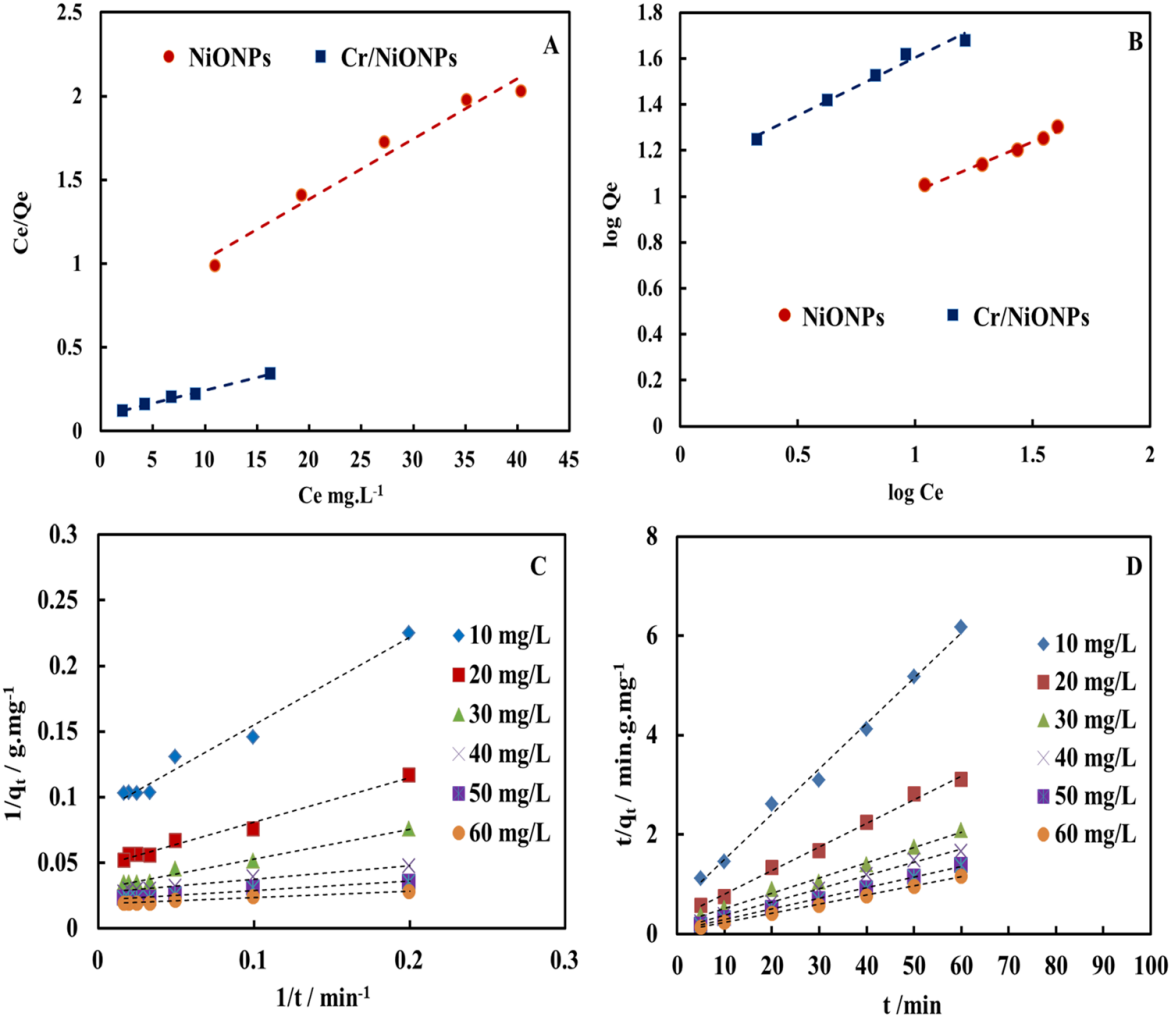
(**A**) and (**B**) Langmuir and Freundlich adsorption isotherms respectively for the adsorption of RR2 on NiONPs and Cr/NiONPs, (**C**) and (**D**) Kinetic study of Cr/NiONPs with RR2 (**C**) pseudo-first-order model and (**D**) pseudo-second-order model respectively.

Adsorption isotherms for adsorption of RR2 dye over each of NiONPs and Cr/NiONPs are shown in Fig. 10 and are presented in Table 1. Freundlich and Langmuir models were investigated for the adsorption of RR2 dye over neat NiONPs and Cr/NiONPs. The adsorption of the RR2 dye on NiONPs and Cr/NiONPs is presented in Fig. 10A,B using the Freundlich and Langmuir models. From the got outcomes that are presented in Table 1, the value of the R^2^ to Langmuir isotherm was greater than the value of the R^2^ for the Freundlich model, and the adsorbed layers number was more than one. In this case, the creation of multilayers of adsorbate on the surface makes the adsorption seems to be more fitted with Langmuir adsorption isotherm^7,9,69^.

**Table 1:** Langmuir and Freundlich Isotherms parameters for RR2 dye adsorption onto Cr/NiONPs.

Adsorbents materials	Langmuir Isotherm			Ferundlich Isotherm		
	q_m_ (mg/g)	KL (L/mg)	R^2^	K_f_ (mg/g)	n	R^2^
NiONPs	27.77	0.054	0.968	3.890	2.318	0.965
Cr/NiONPs	65.78	0.165	0.992	12.566	1.984	0.978

The results of a kinetics study for the adsorption of the RR2 dye onto Cr/NiONPs are shown in Fig. 10C,D and are listed in Table 2. Both the pseudo-first-order model (Fig. 10C) and the pseudo-second-order model (Fig. 10D) were employed as kinetic models for the Cr/NiONPs with RR2. According to the outcomes in Table 2, it was revealed that the correction factor value for the pseudo-first-order kinetic model ranged from 0.978 to 0.982. These values are lower than for the pseudo-second-order kinetic model, which is ranged from 0.994 to 0.999. These outcomes display that a pseudo-second-order kinetic model better describes the RR2 dye adsorption on Cr/NiONPs.

**Table 2:** Kinetic parameters using pseudo-first order and pseudo-second order models of RR2 with Cr/NiONPs.

Conc. of dye mg/L	Pseudo-first order kinetic model				Pseudo-second order kinetic model		
	q_e,exp_ (mg/g)	q_e,cal_ (mg/g)	k_1_ (min^−1^)	R^2^	q_e,cal_ (mg/g)	k_2_ (g/mg min)	R^2^
10	9.720	11.350	6.498	0.978	10.989	0.171	0.994
20	17.791	21.321	6.576	0.982	19.723	0.207	0.996
30	25.786	33.112	6.516	0.981	28.248	0.225	0.998
40	33.604	37.037	3.748	0.982	35.842	0.335	0.999
50	41.015	46.082	3.140	0.954	43.103	0.412	0.999
60	49.593	54.347	2.550	0.978	51.813	0.439	0.999

### Reusability of the used adsorbent and desorption studies

**Figure 11:**
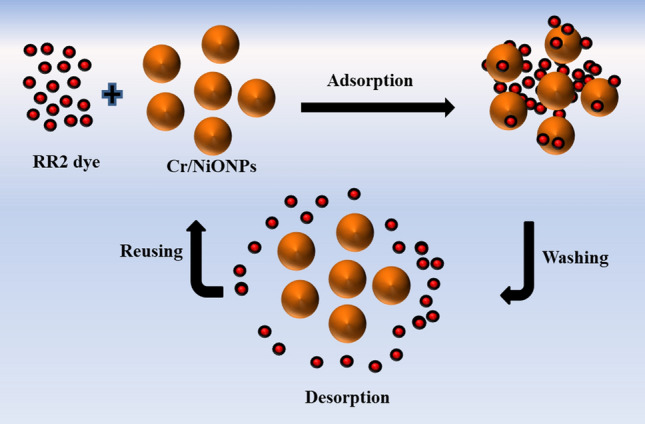
Schematic diagram illustrations of the reusability and desorption of RR2 dye on the Cr/NiONPs.

**Figure 12:**
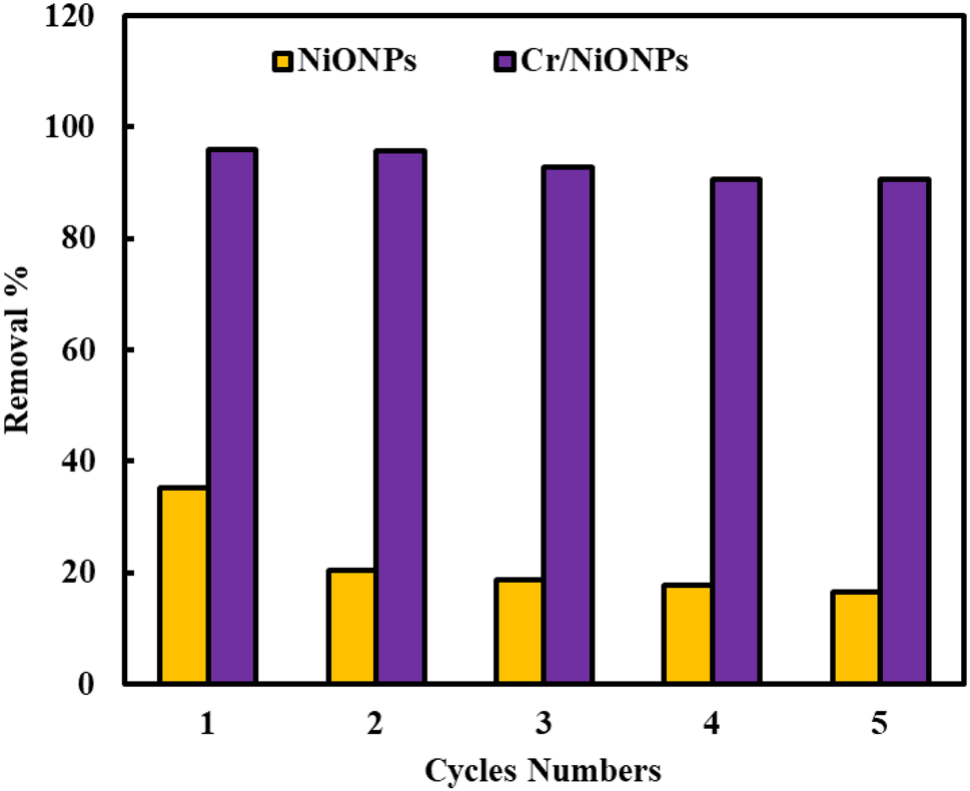
Reusability of NiONPs and Cr/NiONPs for removal RR2 dye (RR2 concentration = 30 mg/L in 100 mL; NPs dosage = 0.1 g; t = 25 °C; pH 6; at 150 rpm).

Desorption studies were performed using NiONPs and Cr/NiONPs as adsorbents and RR2 dyes as adsorbates in order to assess the regeneration and reusability of NiONPs and Cr/NiONPs as adsorbents and assess if they are a material that is economical. Figure 11 shows a schematic diagram of the reusability and desorption of RR2 dye on the Cr/NiONPs. In this experiment, activation and reuse of the used NiONPs and Cr/NiONPs were conducted for five cycles for adsorption/desorption processes and the outcomes are presented in Fig. 12. A series of experiments were performed to investigate the adsorption ability of each of neat NiONPs and Cr/NiONPs applying the same conditions. Desorption of RR2 dye molecules from adsorption sites was performed by immersing the loaded RR2-NiONPs and Cr/NiONPs in a solution of NaOH (0.01 N) for three hours. After that, washing with deionised water was done. The materials were then dried overnight.

Figure 12 shows that after five times of adsorption–desorption, Cr/NiONPs showed high removal efficiency than that neat NiONPs. The results displayed that the removal efficiency of RR2 declined very slightly as the number of reuse cycles increased; which over the five cycles lost just five percent of its initial performance. Thus, the alteration of Cr/NiONPs surface characteristics throughout the adsorption–desorption methods may be the cause of a decline in removal efficiency. These results demonstrate the extremely high recyclability of Cr/NiONPs. This finding makes this catalyst a cost-effective material that makes it a candidate to be used in a wide range of environmental uses, particularly in the treatment of water pollution. Generally, a decline in the adsorption ability of the used material in removing dye molecules is related to the poisoning of the used materials with dye molecules which may result in fewer adsorption positions being available^7,9,70^. As a result of their ease of regeneration and recurrent usage as active adsorbents for water treatment, NiONPs and Cr/NiONPs can be considered to offer promise as environmentally benign adsorbent materials.

Table 3 shows a comparison between our materials (Cr/NiONPs) and other metals doping NiONPs including the type of metal doping NiO and use of these materials to remove which type of pollutants as well as the removal methods they used such as using photocatalytic degradation or adsorption method and the removal percentage.

**Table 3:** Comparison of the metal or metal oxide doping on NiONPs adsorbents for the various dyes removal.

Metal doping NiO NPs adsorbents	Type of pollutants	Removal method	Removal %	References
CuO/NiO	Reactive Red-2 (RR-2) dye	Photocatalytic degradation	96%	^71^
Fe/NiO	Rhodamine B (Rh-B) dye	Photocatalytic degradation	75%	^72^
Mn_3_O_4_/NiO	Methylene blue (MB) dye	Photocatalytic degradation	95%	^73^
Ni/NiO	Aniline blue (AB) dye	Photocatalytic degradation and adsorption	99.5% under UV light 80% in dark condition	^74^
Cr/NiO	Metal ions Pb(II), Cd(II) and Cu(II)	Adsorption	–	^75^
Cr/NiO	Reactive Red-2 (RR-2), And Crystal violet dye (CV)	Adsorption	97% for RR2 15.3% for CV	This work

**Figure 13:**
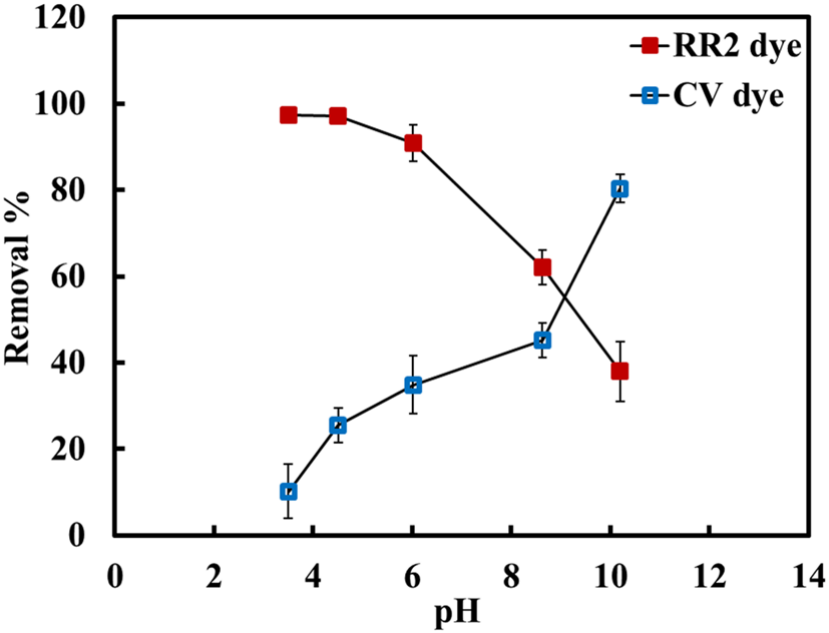
Impact of pH values on adsorption behaviour of RR2 and CV dyes onto Cr/NiONPs.

The effect of pH values on the adsorption of the RR2 and CV dyes using Cr/NiONPs by changing the pH of the dyes between 3 and 10 was investigated as demonstrated in Fig. 13. The results obtained clearly showed that the removal efficiency of RR2 dye increases with the decrease the pH value to reach a maximum of pH 3.5. It was revealed that with decreasing the pH values the R% of this dye greatly increased and reached 98%. In contrast, increasing the pH values shows an increase in the removal efficiency of CV dye. This is due the Cr/NiONPs have a positive surface charge at pH less than 8 and show a net negative charge at pH more than 8.

## Conclusions

In summary, we have developed modified NiONPs which have been functionalised with a dopant of chromium to yield Cr/NiONPs. The co-precipitation technique was successfully used to create nickel oxide nanoparticles and the synthesis of the composite Cr/NiONPs was conducted by the impregnation method. The data displayed that the PZC values for each of neat NiONPs and Cr/NiONPs were at pH 5 and pH 8 respectively. Adsorption of RR2 dye over each of neat NiONPs and Cr/NiONPs was conducted effectively, and the removal efficiency of this dye over Cr/NiONPs was more efficient than that over neat NiONPs. Based on a thorough analysis of the adsorption patterns of various organic dyes, Cr/NiONPs were found to have a very low adsorption capacity against cationic dyes and unique adsorption against anionic dyes. The electrostatic contacts controlled the attraction among the Cr/NiONPs and the dye molecules irrespective of their charge situations. The results of the RR2 dye adsorption equilibrium on free NiONPs and Cr/NiONPs were similarly found to be most compatible with the Langmuir isotherm model. To assess the adsorption process, the kinetics of RR2 adsorption on free NiONPs and Cr/NiONPs were examined. The findings demonstrated that the pseudo-second-order kinetic model could accept the kinetic data. From the obtained results concerned with the reuse of the prepared materials in this study, it can be concluded that these materials are too effective and economically active materials as they showed remarkable activity after five times reuse. We believe that a variety of inorganic NPs like Cu_2_ONPs, ZnONPs, Ag_2_ONPs, TiO_2_NPs, Fe_2_O_3_ and others, will be able to successfully use this kind of functionality which could enable the creation of more effective nanocomposites for dye removal at significantly lower particle concentration.

## Data Availability

The datasets used and/or analysed during the current study are available from the corresponding author upon reasonable request.
